# Optimization of Supercritical CO_2_ Extraction of *Moringa oleifera* Seed Oil Using Response Surface Methodological Approach and Its Antioxidant Activity

**DOI:** 10.3389/fnut.2021.829146

**Published:** 2022-01-21

**Authors:** Xuexiang Chen, Zhuobin Li, Sarah A. Smith, Mingxiu Chen, Hanbin Liu, Jing Zhang, Lirong Tang, Jie Li, Qishan Liu, Xian Wu

**Affiliations:** ^1^School of Public Health, Guangzhou Medical University, Guangzhou, China; ^2^Department of Kinesiology, Nutrition, and Health, Miami University, Oxford, OH, United States

**Keywords:** *Moringa oleifera* seed oil, supercritical CO_2_ extraction, response surface methodology, antioxidant activity, *Moringa oleifera*

## Abstract

Moringa (*Moringa oleifera*) seed oil is an edible vegetable oil rich in unsaturated fatty acids. In this study, the supercritical CO_2_ fluid extraction method was employed to obtain the maximum yield of moringa seed oil. The effects of temperature, time, and pressure, three characteristics of extractions, on the extraction rate of Moringa seed oil were investigated by single factor test and response surface methodological approach. The optimal process conditions of supercritical CO_2_ fluid extraction of moringa seed oil were determined as extraction temperature of 45°C, extraction time of 2.5 h, extraction pressure of 50 MPa, and CO_2_ flow rate of 240 L/h, resulting in a maximum yield of 38.54%. Composition analysis shows that the extracted moringa seed oil is rich in unsaturated fatty acids, including oleic acid, octadecanoic acid, palmitic acid, stearic acid, eicosanoic acid, etc. Furthermore, we found that Moringa seed oil exerted potent antioxidant activities on DPPH and hydroxyl radicals, and its efficacy was comparable to commercial peanut oil and tea oil. Overall, this novel extraction method of moringa seed oil may increase its potential value and application in the food and nutraceutical industries.

## Introduction

Moringa (*Moringa oleifera*), also known as the Drumstick tree, is a Moringa plant found in the Moringa family. It is a perennial tropical deciduous tree that originated in Africa and India (1). Moringa is rich in multiple biological aspects such as protein, amino acids, fibers, vitamins, essential minerals, including potassium, calcium, iron, and zinc, and phytochemicals, including zeatin, quercetin, and kaempferol (2, 3). Moringa seeds contain a relatively large amount of edible oil, especially monounsaturated fatty acids, including oleic, palmitoleic, and gadoleic acids (4–9). In fact, Moringa seed oil contains a higher quantity of unsaturated fatty acids than most common edible oils (4–9). Unsaturated fatty acids are known to be advantageous to human health (10), such as strong antioxidant (11), antitumor (12), and antihyperlipidemic effects (13). In addition, Moringa seed oil is rich in α-tocopherol and δ-tocopherol, and their contents are 45~80 mg/100 g and 0.21~0.53 mg/100 g, respectively (9, 14). Tocopherols are essential nutrients and antioxidants that protect cells from damage (15, 16). Moringa seed oil has been reported to exert potent antioxidant properties, which is at least partially, due to its high content of tocopherols (9, 11). Also, the low content of polyunsaturated fatty acids in Moringa seed oil ensures greater resistance and stability to oxidation (17–19). Because of its good antioxidant potential, Moringa seed oil has been utilized for the preservation of butter oil (20). Blending of butter oil with Moringa seed oil at 2.5–10% level significantly enhanced its monounsaturated fatty acid content and free radical scavenging activity (20). Collectively, Moringa seeds can be used to make a functional edible oil to promote the stability of food products, as well as human health (17–19).

Supercritical CO_2_ fluid extraction (supercritical fluid extraction or SFE) has been applied to a variety of foods due to its low extraction temperature, short extraction time, no solvent residue, high extraction rate, high product quality, energy saving and environmental protection, and good separation effect (21–23). It has shown good application prospects in the fields of food, fine chemical, pharmaceutical industries, as an efficient and environmentally friendly separation and extraction method. Supercritical CO_2_ fluid extraction is particularly suitable for the separation of bioactive substances and natural products, including plant seed oil (24–26). The existing extraction methods of Moringa seed oil, such as ultrasonic assisted extraction, microwave-assisted extraction, rapid solvent extraction, and aqueous enzyme extraction, generally have low extraction rates (27, 28). Specifically, ultrasonic-assisted extraction, microwave-assisted extraction, rapid solvent extraction, and water enzymatic extraction yield 35.85, 35.56, 34.99, and 28%, respectfully, of Moringa seed oil, of which the best is the ultrasonic-assisted extraction method (13). A limitation of these techniques is that they generally require a large amount of organic solvents, some of which are toxic. A prior report has employed supercritical fluid extraction technique to obtain Moringa seed oil and achieved a 39.6% yield (29). However, this study implemented high-pressure CO_2_ fluid (80 MPa), which may limit its application in large-scale production. Thus, it is of importance to optimize the extraction process of Moringa seed oil using milder extraction conditions.

Response surface methodology is a statistical method that has been used to optimize the process parameters and identify the optimal process conditions. Compared with the traditional one-factor-at-a-time approach, response surface methodology is much more efficient. Also, this method considers the interactions among various factors. Thus, the chance of approaching an optimal process condition is much greater compared to the traditional methods (30, 31). The present study employed supercritical CO_2_ fluid extraction as well as the response surface test design on the basis of single factor experiment (14) to investigate the three factors of extraction, temperature, time, and pressure, on the extraction rate of Moringa seed oil. The composition of Moringa seed oil was then determined. Moreover, to further understand its potential food applications and health benefits, *in vitro* antioxidant activity of Moringa seed oil was determined by four factors: DPPH free radical scavenging ability, hydroxyl free radical scavenging ability, superoxide anion free radical scavenging ability, and total antioxidant capacity. The present study provides a scientific basis for the further development and utilization of Moringa and Moringa seed oil.

## Materials and Methods

### Materials and Reagents

Moringa seed (collected in August 2017 in Xiamen, Fujian, China); Moringa seed oil (The extraction was carried out under the best supercritical CO_2_ extraction conditions described in this paper); peanut oil (commercially available); tea oil (commercially available); 1,1-diphenyl-2-picrylhydrazine radical (DPPH) and T-AOC total antioxidant kit were purchased from Nanjing Jiancheng Institute of Biology (Nanjing, China). vitamin E standard (Macklin, Shanghai, China); Gas chromatography–mass spectrometry (GC-MS, Voyager, Thermo Fisher, Waltham, MA, USA); food-grade CO_2_ and food-grade N_2_ (Guangzhou Yuejia Gas, Guangzhou, China); bromine-based carbon tetrachloride; concentrated sulfuric acid; trichloromethane; anhydrous ethanol; hydrochloric acid; ferrous sulfate; pyrogallol (pyrogallol) acid); 30% hydrogen peroxide; salicylic acid (chemical reagents are all analytical pure).

### Supercritical CO_2_ Extraction

All supercritical-CO_2_ extraction trials were carried out in a SFE-2 ASI Supercritical Extraction Apparatus (Universal Analytical Testing Instruments Co., Ltd., Hong Kong, China), according to the manufacturer's instructions. The purity of CO_2_ is 99%. Briefly, liquid CO_2_ supplied from a gas cylinder was cooled by ethanol to 5°C before being pressurized to the desired pressure and passed into the device; the entire device was also pre-pressurized. For each experiment, ~15 g crushed and dried Moringa seed powder was loaded into a steel cylinder. The loaded cylinder was then introduced into the extraction vessel, and CO_2_ was allowed into the vessel. During the extraction process, the extraction pressure, extraction temperature, and CO_2_ flow rate were controlled by adjusting the valves on the front panel. When the scheduled time was achieved, the extraction vessel was depressurized and the oil was collected from the separation vessel. The oil obtained under the optimum condition was used for the variety of tests that follow. The extraction rate of Moringa seed oil is calculated as the percentage of the extracted Moringa seed oil to the total amount of raw materials.


$$\begin{array}{l} \text{Extraction rate }\left(\%\right)=\frac{{\text{P}}_{1}}{{\text{P}}_{0}}*100\% \end{array}$$

Where P_0_ represents the total amount of raw materials, while P_1_ represents the extracted Moringa seed oil.

### Effect of Extraction Temperature on the Yield of Moringa Seed Oil

The extraction conditions of Moringa seed powder were: 15 g of Moringa seed powder, the extraction time was 2 h, the extraction pressure was 35 MPa, and the CO_2_ flow rate was 4 L/min. For supercritical CO_2_ extraction under five levels of conditions, the extraction temperatures were selected to be 40, 45, 50, 55, and 60°C to examine the effect of extraction temperature on the extraction rate of Moringa seed oil.

### Effect of Extraction Time on the Yield of Moringa Seed Oil

The extraction conditions of Moringa seed powder were: 15 g of Moringa seed powder, the extraction pressure was 45 MPa, the extraction temperature was 45°C, the CO_2_ flow rate was 4 L/min. The extraction time was 1, 2, 3, 4, and 5 h respectively, to examine the effect of extraction time on the extraction rate of Moringa seed oil.

### Effect of Extraction Pressure on the Yield of Moringa Seed Oil

The extraction conditions of Moringa seed powder were: 15 g of Moringa seed powder, the extraction time was 2 h, the extraction temperature was 50°C, the CO_2_ flow rate was 4 L/min, and the supercritical CO_2_ extraction was performed under 5 levels of pressure, 30, 35, 40, 45, and 50 MPa, to examine the effect of extraction pressure on the extraction rate of Moringa seed oil.

### Response Surface Design Test of Supercritical CO_2_ Extraction Process

On the basis of single factor experiment, according to Box-Behnken design principle (14), the extraction pressure, extraction time, and extraction temperature are selected as independent variables, and the extraction rate of Moringa seed oil is the response value. Design-Expert 8.0.5 software was used to perform response surface analysis to optimize the extraction conditions. The response surface test factor coding and level design are shown in Table 1.

**Table 1:** The factor and level of response surface methodology in the extraction of Moringa seed oil by supercritical CO_2_.

**Level**	**Factors**		
	**A Time (h)**	**B Temperature (°C)**	**C Pressure (MPa)**
−1	1	40	40
0	2	45	45
1	3	50	50

### Component Analysis

Fifteen drops of Moringa seed oil and 2 mL of 2% hydrochloric acid methanol solution were added into a 5 mL brown ampoule, and the ampoule was shaken for 30 s and seal the mouth of ampoule bottle with alcohol blowtorch at high temperature. At a high temperature, the mouth of the ampoule was sealed with a blowtorch, and immediately put it in a blast drying oven at 100°C for 40 min. Moringa seed oil was analyzed by GC-MS: TG WaxMS GC column (30 m × 0.25 mm × 0.25 μm), carrier gas was helium, the purity of helium was 99.99%, flow rate was 1 mL/min, inlet temperature was 285°C. Heating conditions: 50°C (2 min) → 260°C (30 min). Mass spectrometry conditions: ion source temperature was 230°C, EI source electron energy was 70 eV, split ratio was 50:1, sample volume was 1.0 μL, scanning range was 45~450 amu (31).

### Antioxidant Assays

#### DPPH Free Radical Scavenging Assay

The antioxidant capacity of Moringa seed oil was compared with peanut oil and tea seed oil (5~35 mg/mL in chloroform), and vitamin E (1~5 mg/mL in chloroform), which was used as the control. DPPH free radical scavenging assay was performed as reported previously (31). Briefly, 4 mL of the chloroform extract of each oil sample and 1 mL of the 0.2 mmol/L DPPH solution were added to the same test tube with a stopper and shaken well. The tubes were let stand tightly sealed at room temperature for 30 min. Chloroform was used as the reference solution at 517 nm. The absorbance was measure at the wavelength and measure three times in parallel.


$$\begin{array}{c} \text{DPPH free radical scavenging percentage }\left(\%\right)\\ \;=\frac{1-\left({\text{A}}_{\text{s}}-{\text{A}}_{\text{sb}}\right)}{{\text{A}}_{\text{c}}}*100\% \end{array}$$

Where A_s_ is the absorbance of DPPH solution after adding 4 mL of extract; A_sb_ is the absorbance of 4 mL of extract + 1 mL of solvent (chloroform); A_c_ is the absorbance of 4 mL of solvent (chloroform) + 1 mL DPPH.

#### Hydroxyl Radical (HO·) Scavenging Assay

Hydroxyl radical (HO·) scavenging assay was performed as reported previously (32). 1~5 mg/mL Moringa seeds Oil, peanut oil, tea oil solution were dissolved in absolute ethanol 0.2 mL of oil sample solution with different concentration gradient, 0.6 mL of 8.0 mmol/L FeSO_4_ solution, 0.5 mL of 20 mmol/L H_2_O_2_ solution and 2.0 mL of 3.0 mmol/L salicylic acid solution were mixed together; After the reaction, at the wavelength of 510 nm, the specific tone of ethanol was zero, the supernatant in the EP tube was taken to determine the absorbance A1, the A2 value was determined with ethanol instead of 20 mmol/L H_2_O_2_, and the A0 value was determined with ethanol instead of the sample solution for 3 times in parallel.


$$\begin{array}{c} \text{Hydroxyl radical scavenging percentage }\left(\%\right)\\ \;=\frac{{\text{A}}_{0}-\left({\text{A}}_{1}-{\text{A}}_{2}\right)}{{\text{A}}_{0}}*100\text{\%} \end{array}$$

Where A_1_ is the absorbance value of the sample to be tested; A_2_ is the absorbance value of the H_2_O_2_ reagent blank; A_0_ is the absorbance value of the sample blank.

#### Superoxide Radical (O2·-) Scavenging Activity Assay

Superoxide radical (O2·-) scavenging assay was performed as reported previously (33). One milliliter of horseradish seed oil, peanut oil, tea oil, and vitamin E samples were prepared, 5 mL of Tris HCl solution with pH of 8.2 and 50 mmol/L pyrogallol solution were added, distilled water was used as the blank control, and its absorbance value was measured at 325 nm every 30 s.


$$\begin{array}{c} \text{O}2\cdot -\text{ scavenging rate }\left(\%\right)=\left[1-\frac{\left({\text{A}}_{1}-{\text{A}}_{2}\right)}{{\text{A}}_{0}}\right]*100\% \end{array}$$

Where A_1_ is the absorbance value of the sample plus Tris-HCL solution and pyrogallol; A_2_ is the absorbance value of the sample plus Tris-HCL solution and distilled water; A_0_ is the absorbance value of the sample blank.

#### Total Antioxidant Capacity Assay

The total antioxidant capacity was measured according to the manufacturer's instructions (34). The absorbance of each tube was measured at the wavelength of 520 nm. At 37°C, when the absorbance (A) of the reaction system increases by 0.01 per milliliter of sample per minute, it is a unit of total antioxidant capacity (U), and the total antioxidant capacity is calculated by the following formula.

### Statistical Analysis

All results are expressed as mean ± standard deviation (SD). Statistical significance between groups was determined by a Student's two-tailed *t*-test (between two groups) or an analysis of variance (ANOVA) (between more than two groups). A *p* < 0.05 was considered statistically significant.

## Results and Discussion

### Effect of Extraction Temperature on the Yield of Moringa Seed Oil

**Figure 1:**
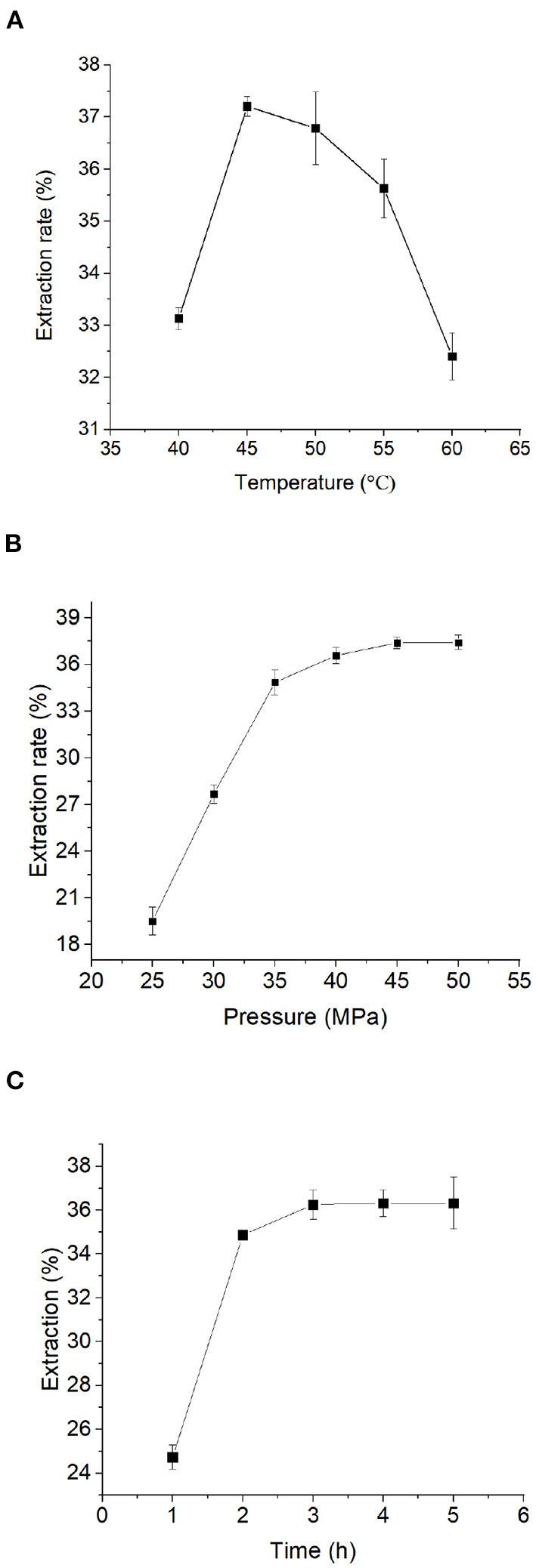
Single factor test: Influence of **(A)** temperature, **(B)** time, and **(C)** pressure on the extraction rate of Moringa seed oil. Data were expressed as mean ± SD.

To optimize the extraction conditions of Moringa seed oil, we first sought to determine the optimal extraction temperature. According to the existing literature (35), the extraction temperature range was chosen between 40 and 60°C, at 5 levels for supercritical CO_2_ extraction. It can be seen from Figure 1A that the effect of extraction temperature on the extraction rate of Moringa seed oil was a curve that first rises and then falls. When the extraction temperature was around 40~45°C, the extraction rate of Moringa seed oil increased rapidly as the temperature rose. When the extraction temperature reached 45°C, the extraction rate of Moringa seed oil was the highest among all the five temperature points. When the extraction temperature was between 45 and 60°C, the extraction rate of Moringa seed oil decreased as the temperature increased. In summary, from the perspective of energy saving and efficiency improvement, the optimal extraction temperature for supercritical CO_2_ extraction of Moringa seed oil is around 45~50°C.

### Effect of Extraction Time on the Yield of Moringa Seed Oil

Next, we sought to determine the optimal extraction time for Moringa seed oil. In this experiment, the extraction temperature was 50°C as determined in prior experiment, the extraction pressure was 35 MPa, and the extraction time was 1, 2, 3, 4, and 5 h for supercritical CO_2_ extraction. It can be seen from Figure 1B that at 1–2 h the increase was significant, but when the extraction time was >2 h, the extraction rate of Moringa seed oil reached a plateau. Therefore, from the perspective of energy saving and efficiency improvement, the optimal extraction time for supercritical CO_2_ extraction of Moringa seed oil is set at 2 h.

### Effect of Extraction Pressure on the Yield of Moringa Seed Oil

Next, the effect of pressure on the extraction rate of Moringa seed oil was determined. The extraction temperature was 50°C, the extraction time was 2 h, as determined in prior experiments, and the extraction pressure was 25, 30, 35, 40, 45, 50 MPa in this experiment. It can be seen from Figure 1C that the effect of the pressure on the extraction rate of Moringa seed oil was similar to that of that of the extraction time. Below 35 MPa, as the pressure increased, the extraction rate of Moringa seed oil increased, and the extracted Moringa seed oil was light yellow, but the color was slightly different (data not shown). When the extraction pressure was >45 MPa, the extraction rate of Moringa seed oil reached a plateau, but the color of Moringa seed oil gradually deepened. At 50 MPa, the Moringa seed oil was a dark yellow oily substance and this might be because of some other substances were extracted together under such a high pressure. Collectively, from the perspective of energy saving and efficiency improvement, the optimal extraction pressure for supercritical CO_2_ extraction of Moringa seed oil is set at 45 MPa.

### Response Surface Analysis

To conduct the single factor test of supercritical CO_2_ extraction of Moringa seed oil, the extraction temperature of 40, 45, and 50°C, the extraction time of 1, 2, and 3 h, and the extraction pressure of 40, 45, and 50 MPa were used as the independent variable and the extraction rate of Moringa seed oil was the response value. The response surface test design and results are shown in Table 2. Design-expert V8.0.6 analysis software was used to perform multiple regression fitting on the experimental data in Table 2 to obtain the secondary extraction rate Y of Moringa seed oil against A (extraction time), B (extraction temperature), and C (extraction pressure). The multinomial regression equation was:


$$\begin{array}{l} \text{Y }=\;38.22-2.52A-0.21B+0.94C+0.48AB-0.9AC\\ \;+\;0.14BC-2.92A^{2}-1.26B^{2}+0.41C^{2} \end{array}$$

In order to test the reliability of the regression equation and determine the influence of various factors on the extraction rate of Moringa seed oil, variance analysis was carried out on the regression equation. Analysis of variance results (Table 3) showed that the regression model *p*-value was 0.0033, < 0.05, indicating that the regression model was significant. The fitting error was 0.1019, which was >0.05, indicating that the fitting error was not significant, and the regression model fitted well with the actual situation. According to the value of F, the influence of each factor on the extraction rate of Moringa seed oil was a > c > b, that is, extraction time > extraction pressure > extraction temperature.

**Table 2:** Extraction rate of Moringa seed oil by supercritical CO_2_ with varying time, temperature, and pressure using response surface methodological approach.

**Level**	**A** **Time (h)**	**B** **Temperature (°C)**	**C** **Pressure (MPa)**	**Extraction** **rate (%)**
1	3	45	40	37.56
2	1	45	40	31.74
3	2	45	45	38.61
4	2	40	50	37.90
5	1	50	45	29.98
6	3	45	50	37.88
7	3	40	45	37.14
8	2	50	50	38.45
9	2	45	45	38.02
10	3	50	45	36.98
11	2	45	45	38.02
12	2	50	40	36.54
13	2	40	40	36.56
14	1	40	45	32.05
15	1	45	50	35.66

**Table 3:** Variance analysis of regression model on the extraction conditions of Moringa seed oil using response surface methodological approach.

**Source**	**Sum of**	**df**	**Mean**	***F*** **-value**	***P*** **-value**	**Significant**
	**squares**		**squares**			
model	99.53	9	11.06	16.47	0.0033	**
A (Time)	50.65	1	50.65	75.44	0.0003	**
B (Temperature)	0.36	1	0.36	0.54	0.4962	NS
C (Pressure)	7.01	1	7.01	10.44	0.0232	*
AB	0.91	1	0.91	1.36	0.2964	NS
AC	3.24	1	3.24	4.83	0.0794	NS
BC	0.081	1	0.081	0.12	0.7421	NS
A^2^	31.39	1	31.39	46.76	0.001	**
B^2^	5.89	1	5.89	8.78	0.0314	*
C^2^	0.62	1	0.62	0.92	0.3814	NS
Residual	3.36	5	0.67			
Lack of fit	3.13	3	1.04	8.98	0.1019	
Pure error	0.23	2	0.12			
Cor total	102.89	14				

* and ** denote significant differences p < 0.05 and < 0.01, respectively.

**Figure 2:**
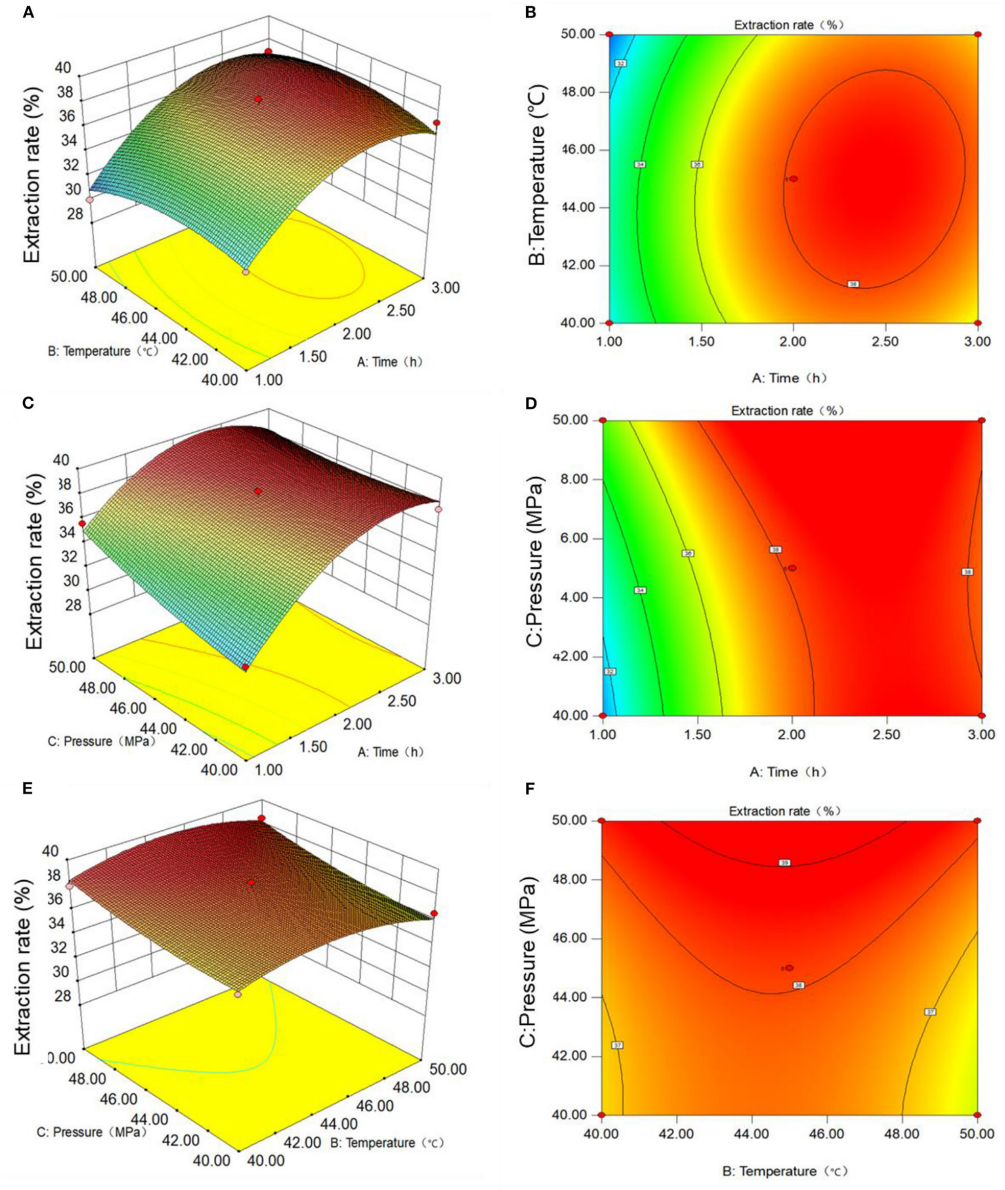
Response surface and contour plots showing the interactive effect of different extraction conditions on the extraction efficiency of Moringa seed oil: **(A,B)** temperature and time; **(C,D)** temperature and pressure; **(E,F)** time and pressure.

In order to more intuitively show the influence of the three factors on the extraction rate of Moringa seed oil, the stereogram and contour map of response surface between each factor and corresponding value were created, as shown in Figures 2A,B. Figure 2A shows the response surface and contour plots for the effects of extraction temperature and time at a constant extraction pressure of 45 MPa on the extraction rate of Moringa seed oil. With the increase of extraction temperature and time, the extraction rate of Moringa seed oil shows a trend from low to high, that is, under specific extraction temperature and time, the extraction rate of Moringa seed oil has a maximum value, the extraction rate was 38%, which is at the top of response surface map. Through the contour shape, we can see the strong and weak relationship of interaction effect. The closer the shape is to the circle, the less significant the interaction effect is. It can be seen from contour lines in the right panel of Figure 2B that the graph is close to a circle, indicating that the interaction between the two factors, extraction temperature and time, is not very significant.

Similarly, Figure 2C shows the response surface and contour plots for the effects of temperature and pressure at a constant extraction time of 2 h on the extraction rate of Moringa seed oil. With the increase of extraction temperature and pressure, the extraction rate of Moringa seed oil is increasing from low to high. It can also be seen that the extraction temperature has a great influence on the extraction rate of Moringa seed oil. Under specific extraction temperature and pressure, the extraction rate of Moringa oil has a maximum value, which exists at the top of response surface. The contour line in the right panel of Figure 2D is not circular nor oval, but parallel line, indicating that the interaction between extraction temperature and extraction pressure is insignificant.

Lastly, the interaction between the extraction time and pressure was shown in Figure 2E. It can be seen that when the extraction temperature remains unchanged at 45°C, with the increase of extraction time and pressure, the extraction rate of Moringa seed oil shows a trend from low to high. Under the appropriate extraction time and extraction pressure, the extraction rate of Moringa seed oil has a maximum value, which is at the top of response surface diagram. The contour line in the right panel of Figure 2F is a parallel line, indicating that the interaction between extraction time and extraction pressure is influence.

Comprehensive analysis of the above results was then performed. The optimal extraction conditions of supercritical CO_2_ extraction of Moringa seed oil were as follows: extraction temperature of 45.12°C, extraction time of 2.28 h, and extraction pressure of 50 MPa, and the highest extraction rate would be 39.78%. Considering the convenience of practical operation, the optimal extraction conditions of supercritical CO_2_ extraction of Moringa seed oil were modified to extraction temperature of 45°C, extraction time of 2.5 h and extraction pressure of 50 MPa on the basis of the theoretical value obtained from the regression equation. Then we performed the extraction under the optimal conditions and the results are shown in Table 4. It can be seen that the actual extraction rate of Moringa seed oil is 38.54%, which is only 1.24% lower compared with the theoretical optimum value of 39.78%. The results also indicated that the model was reliable and reproducible in predicting supercritical CO_2_ extraction of Moringa seed oil.

**Table 4:** Results of the verification test.

**No**.	**Temperature**	**Time**	**Pressure**	**Extraction**	**Average**
	**(**°**C)**	**(h)**	**(MPa)**	**rate (%)**	**(%)**
1	45	2.5	50	39.0	38.54 ± 0.39
2	45	2.5	50	38.2	
3	45	2.5	50	38.4	

Ultrasonic assisted, microwave-assisted, rapid solvent, and water enzymatic extraction methods yielded an extraction rate between 28 and 35.85% (13), which are generally lower than the optimal extraction rate of supercritical CO_2_ extraction. Duan et al. used ultrasonic assisted solvent extraction to obtain Moringa seed oil, and the optimal extraction rate was 36.3% (31), which was also slightly lower than the method we developed in the present study. A prior study using an extraction condition of 80 MPa and 57°C of supercritical fluid achieved a 39.6% yield of Moringa seed oil (29). In contrast, we implemented a milder extraction condition of 50 MPa and 45°C, and the optimal yield was only 1.06% lower compared to the report (29), suggesting that the extraction conditions optimized in the present might be a more economic and convenient supercritical fluid extraction method for industrial scale production.

### GC-MS Analysis of Moringa Seed Oil

**Figure 3:**
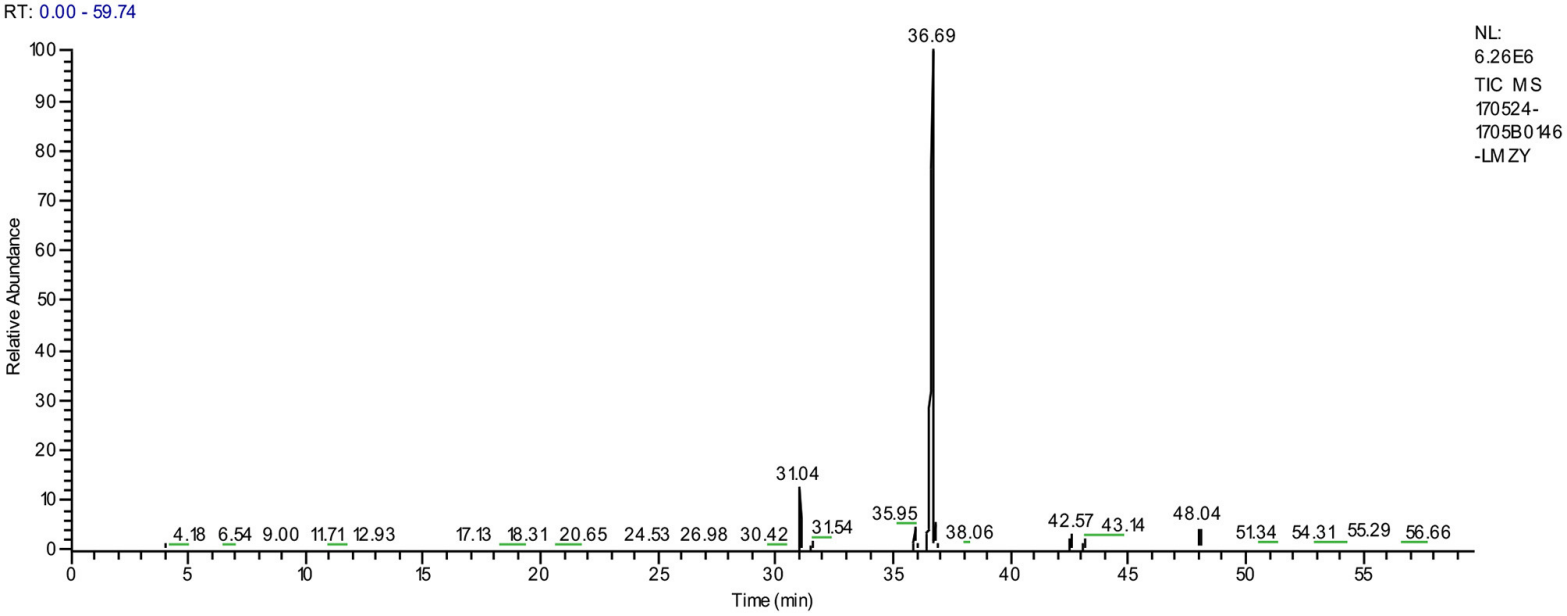
GC-MS total ion chromatogram of Moringa seed oil.

The composition of Moringa seed oil was determined by GC-MS. As shown in Figure 3, Table 5 that the oil mainly contains fatty acids, which account for 98.16%, of which 0.92% is β-sitosterol, stigmasterol and rapeseed oil sterol, and 85.43% is oleic acid. Natural oleic acid has a protective effect on blood vessels, and plays an important role in the metabolic process of humans and animals (36). However, the oleic acid synthesized by human body cannot meet the required needs of the body, and the most of the oleic acid needs to be ingested by humans from food. Therefore, edible oils with high oleic acid content, such as olive oil and Moringa seed oil, are beneficial to health (37).

**Table 5:** Composition of Moringa seed oil.

**No**.	**Name of compound**	**Molecular**	**Relative**
		**formula**	**content (%)**
1	Oleic acid	C_18_H_34_O_2_	85.4
2	Octadecanoic acid	C_18_H_36_O_2_	3.81
3	Palmitic acid	C_16_H_32_O_2_	2.31
4	10,13-Dimethyltetradecanoic acid	C_16_H_32_0_2_	2.42
5	Myristic acid	C_14_H_28_O_2_	0.03
6	Stearic acid	C_18_H_36_O_2_	2.65
7	Eicosanoic acid	C_20_H_40_O_2_	1.51
8	β- Sitosterol	C_29_H_50_O	0.3
9	Stigmasterol	C_29_H_48_O	0.48
10	Rapeseed oil sterol	C_28_H_48_O	0.14

### Antioxidant Activity of Moringa Seed Oil

#### Scavenging Effect of Moringa Seed Oil on DPPH Free Radical

**Figure 4:**
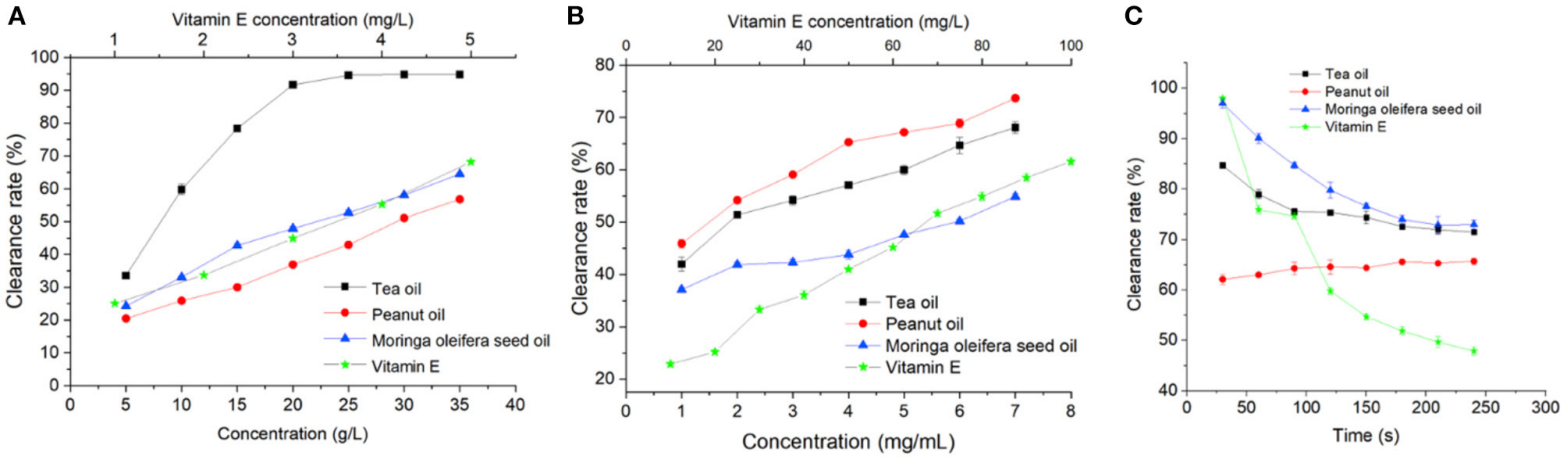
Antioxidant activities of Moringa seed oil, tea oil, peanut oil, and vitamin E: **(A)** DPPH free radical scavenging assay, **(B)** hydroxyl radical scavenging assay, and **(C)** superoxide radical (O2·-) scavenging activity assay. Data were expressed as mean ± SD.

Antioxidant and free radical scavenging activity of Moringa seed oil was first determined by DPPH assay. As shown in Figure 4A, vitamin E showed a strong ability to remove DPPH radicals. Moringa seed oil, peanut oil, and tea oil also effectively removed DPPH radicals, and DPPH free radical scavenging rate was positively correlated with the concentration of the oils. Interestingly, the DPPH free radical scavenging activity of tea oil was much higher than that of Moringa oil and peanut oil. The EC_50_ of tea oil is 8.39 mg/mL, while Moringa seed oil and peanut oil have EC_50_ of 22.94 and 29.98 mg/mL, respectively (Supplementary Table S1).

#### Scavenging Effect of Moringa Seed Oil on Hydroxyl Radical

Next, hydroxyl radical scavenging activities of Moringa seed oil was determined. As shown in Figure 4B, all the oils and vitamin E exert hydroxyl radical scavenging effect in a dose dependent manner. In contrast to the DPPH scavenging assay, Moringa seed oil is the strongest among all the oils. As shown in Supplementary Table S2, the EC_50_ of Moringa seed oil, peanut oil, and tea oil is 1.21, 5.71, and 2.28 mg/mL, respectively.

#### Superoxide Anion Radical Scavenging Activities of Moringa Seed Oil

In addition to DPPH free radicals and hydroxyl radicals, superoxide anion radical (O2·-) is one of the most destructive reactive oxygen species. As shown in Figure 4C, Supplementary Table S3, in the first 60 s, vitamin E exhibited the fastest clearance rate, reaching 98.1%, but then its clearance rate dropped quickly. Among all three oils, Moringa seed oil showed the strongest scavenging capacity of superoxide anion radicals, and the maximum scavenging rate reached 97%. With the increase of time, its inhibition rate decreased continuously, but it was still slightly higher than that of peanut oil.

#### Determination of Total Antioxidant Capacity

Lastly, the total antioxidant capacity of Moringa seed oil was determined. As shown in Table 6, the total antioxidant capacity of vitamin E is the highest, which was 54.6 U/mL, whereas Moringa seed oil, tea oil, and peanut oil had a total antioxidant capacity of 3.22, 2.98, and 0.89 U/mL, respectively. Collectively, the DPPH free radical, hydroxyl radical, and superoxide anion radical scavenging, and total antioxidant capacity assays confirm that the Moringa seed oil extracted has potent antioxidant property, comparable to other common edible oils.

**Table 6:** Total antioxidant capacity of different edible oils and vitamin E.

	**Moringa seed oil**	**Tea oil**	**Peanut oil**	**Vitamin E**
T—AOC (U/mL)	3.22 ± 0.09	0.89 ± 0.02	2.98 ± 0.01	54.6 ± 0.37

## Conclusion

In this study, Moringa seed oil was obtained by supercritical CO_2_ extraction combined with response surface methodological approach. The optimal process conditions of supercritical CO_2_ fluid extraction of moringa seed oil were determined as extraction temperature of 45°C, extraction time of 2.5 h, extraction pressure of 50 MPa, and CO_2_ flow rate of 240 L/h, resulting in a maximum yield of 38.54%. In addition, the fatty acid composition of Moringa seed oil was analyzed by GC-MS. Moreover, *in vitro* antioxidant activity of Moringa seed oil was evaluated using multiple radical scavenging assays. It was found that Moringa seed oil had higher DPPH radical scavenging capacity than commercial peanut oil, and its hydroxyl radical and superoxide anion radical scavenging capacity was higher than both commercial tea oil and peanut oil. The total antioxidant capacity of Moringa seed oil was also greater than commercial tea oil and peanut oil. Overall, this study provides a scientific basis for the development and application of Moringa seed oil in functional food, nutraceutical, and cosmetic products.

## Data Availability Statement

The original contributions presented in the study are included in the article/Supplementary Material, further inquiries can be directed to the corresponding author/s.

## Author Contributions

XC and XW designed the experiment. MC, HL, and XC conducted the experiments. ZL, XC, LT, JL, and QL performed the experimental analysis, collection, and analysis of the data. ZL, SS, JZ, XC, and XW wrote and revised the manuscript. All authors contributed to the article and approved the submitted version.

## Funding

This work was supported by General project of Basic and applied basic Research in Guangzhou (2060206); Natural Science Foundation of Guangdong basic and applied basic research foundation (2021A1515010965); Natural Science Foundation of Guangdong basic and applied basic research foundation ([2018]105), Laboratory opening project of Guangzhou Medical University (PX-1020423).

## Conflict of Interest

The authors declare that the research was conducted in the absence of any commercial or financial relationships that could be construed as a potential conflict of interest.

## Publisher's Note

All claims expressed in this article are solely those of the authors and do not necessarily represent those of their affiliated organizations, or those of the publisher, the editors and the reviewers. Any product that may be evaluated in this article, or claim that may be made by its manufacturer, is not guaranteed or endorsed by the publisher.

## Abbreviations

SFE: supercritical fluid extraction
DPPH, 2: 2-Diphenyl-1-picrylhydrazyl.

## References

[1] Chen D Zhang X Zhang H. A new functional edible oil – *Moringa oleifera* seed oil. Guangdong Agric Sci. (2008) 218:17–8. 10.16768/j.issn.1004-874x.2008.05.001

[2] Liu F Wang M Zhao Y Xu X Pan S. Extraction of functional components from *Moringa oleifera* and development of *Moringa oleifera*-based products. Food Sci. (2015) 36:282–6. 10.7506/spkx1002-6630-201519051

[3] Nadeem M Imran M. Promising features of *Moringa oleifera* oil: recent updates and perspectives. Lipids Health Dis. (2016) 15:1–8. 10.1186/s12944-016-0379-0 27931216 PMC5146848

[4] Famurewa AC Aja PM Nwankwo OE Awoke JN Maduagwuna EK Aloke C. *Moringa oleifera* seed oil or virgin coconut oil supplementation abrogates cerebral neurotoxicity induced by antineoplastic agent methotrexate by suppression of oxidative stress and neuro-inflammation in rats. J Food Biochem. (2019) 43:e12748. 10.1111/jfbc.12748 31353570

[5] Gharsallah K Rezig L Msaada K Chalh A Soltani T. Chemical compositionand profile characterization of *Moringa oleifer*a seed oil. S Afr J Bot. (2021) 137:475–82. 10.1016/j.sajb.2020.11.014

[6] Wang L Xu Y Liu Y. The research progress of active components and its physiological functions of *Moringa oleifera* seeds. Food Res Dev. (2019) 40:190–5. 10.3969/j.issn.1005-6521.2019.04.034

[7] Lin L Gu Y Cui H. Moringa oil/chitosan nanoparticles embedded gelatin nanofibers for food packaging against *Listeria monocytogenes* and *Staphylococcus aureus* on cheese. Food Packag Shelf Life. (2019) 19:86–93. 10.1016/j.fpsl.2018.12.005

[8] Deng W Wang M Lv Q. Study on composite enzymatic extraction and *in vitro* anti-oxidative of *Moringa oleifera* seed oil. Cereals Oils. (2019) 32:67–72. 10.3969/j.issn.1008-9578.2019.12.017

[9] Leone A Spada A Battezzati A Schiraldi A Aristil J Bertoli S. *Moringa oleifera* seeds and oil: characteristics and uses for human health. Int J Mol Sci. (2016) 17:2141. 10.3390/ijms17122141 27999405 PMC5187941

[10] Shahidi F Ambigaipalan P. Omega-3 polyunsaturated fatty acids and their health benefits. Annu Rev Food Sci Technol. (2018) 9:345–81. 10.1146/annurev-food-111317-095850 29350557

[11] Peng X Li J Zhang M. Effect of *Moringa oleifera* oil on life-span, superoxide dismutase activity and maleic dialdehyde content of drosophila melanogaster. Hubei Agric Sci. (2009) 48:555–7. 10.3969/j.issn.0439-8114.2009.03.014

[12] Elsayed EA Sharaf-Eldin MA Wadaan M. In vitro evaluation of cytotoxic activities of essential oil from *Moringa oleifera* seeds on HeLa, HepG2, MCF-7, CACO-2 and l929 cell lines. Asian Pac J Cancer Prev. (2015) 16:4671–5. 10.7314/APJCP.2015.16.11.4671 26107222

[13] Yu J. Study on Extraction Method and Hypolipidemic Effect on Moringa oleifera Seed Oil. Kunming: Kunming Medical University (2009).

[14] Wang T Yang M Yang F Du P. Determination and multivariate statistical analysis of functional components of *Moringa oleifera* seed oil. Food Sci. (2020) 41:145–50. 10.7506/spkx1002-6630-20190828-314

[15] Di Vincenzo Tana C El Hadi H Pagano C Vettor R Rossato M. Antioxidant, anti-inflammatory, and metabolic properties of tocopherols and tocotrienols: clinical implications for vitamin e supplementation in diabetic kidney disease. Int J Mol Sci. (2019) 20:5101. 10.3390/ijms20205101 31618817 PMC6834186

[16] Yang C Luo P Zeng Z Wang H Malafa M Suh N. Vitamin E and cancer prevention: studies with different forms of tocopherols and tocotrienols. Mol Carcinog. (2020) 59:365–89. 10.1002/mc.23160 32017273 PMC7255062

[17] Dollah S Chai KF Abdulkarim SM Ghazali HM. Comparative study of table margarine prepared from *Moringa oleifera* seed oil-palm stearin blend and commercial margarines: composition, thermal, textural properties. Eur J Lipid Sci Technol. (2020) 122:1900428. 10.1002/ejlt.20190042825855820

[18] Jamieson GS. Ben (moringa) seed oil. Oil Soap. (1939) 16:173–4. 10.1007/BF02543375

[19] Ogunsina BS Indira TN Bhatnagar AS Radha C Debnath S Gopala Krishna AG. Quality characteristics and stability of Moringa oleifera seed oil of Indian origin. J Food Sci Technol. (2014) 51:503–10. 10.1007/s13197-011-0519-5 24587525 PMC3931868

[20] Nadeem M Abdullah M Hussain I. Improvement of the oxidative stability of butter oil by blending with *M oringa oleifera* oil. J Food Process Preserv. (2014) 38:1491–500. 10.1111/jfpp.12108

[21] Ahangari H King JW Ehsani A Yousefi M. Supercritical fluid extraction of seed oils – a short review of current trends. Trends Food Sci Technol. (2021) 111:249–60. 10.1016/j.tifs.2021.02.066

[22] Picot-Allain C Mahomoodally MF Ak G Zengin G. Conventional versus green extraction techniques — a comparative perspective. Curr Opin Food Sci. (2021) 40:144–56. 10.1016/j.cofs.2021.02.009

[23] Villacís-Chiriboga J Vera E Van Camp J Ruales J Elst K. Valorization of byproducts from tropical fruits: a review, Part 2: applications, economic, and environmental aspects of biorefinery via supercritical fluid extraction. Compr Rev Food Sci Food Saf. (2021) 20:2305–31. 10.1111/1541-4337.12744 33864344

[24] Ren F Han F Shi L Bao S Shi S. Application of supercritical CO2 fluid extraction technology of vegetable oil. China Oils and Fats. (2010) 35:14–9.

[25] Liu Y. Application of Supercritical Fluid Extraction on Removing Plasticizers in Spores of Ganoderma lucidum. Guangzhou: South China Agriculture University (2017).

[26] Chen W Wang Z. Application of supercritical extraction. China West Cereals Oils Technol. (2003) 28:38–40. 10.3969/j.issn.1007-6395.2003.06.016

[27] Lv Q Xiang Z Xu X Ji D Deng L Song D. Quality analysis of *Moringa oleifera* seed oil extracted by different methods. Food Sci Technol. (2018) 43:226–31. 10.13684/j.cnki.spkj.2018.03.043

[28] Zhang J Wang S Wang X Li D. Extraction of *Moringa oleifera* oil by the method of simultaneous distillation extraction and analysis of its constituent. J Fujian Agric Forestry Univ. (2018) 47:367–72. 10.13323/j.cnki.j.fafu(nat.sci.).2018.03.016

[29] Belo YN Al-Hamimi S Chimuka L Turner C. Ultrahigh-pressure supercritical fluid extraction and chromatography of *Moringa oleifera* and *Moringa peregrina* seed lipids. Anal Bioanal Chem. (2019) 411:3685–93. 10.1007/s00216-019-01850-x 31053955 PMC6571088

[30] Liyana-Pathirana C Shahidi F. Optimization of extraction of phenolic compounds from wheat using response surface methodology. Food Chem. (2005) 93:47–56. 10.1016/j.foodchem.2004.08.050

[31] Duan Q Liu F Luo J Ma L Wang Y Zhang Z. Extraction of *Moringa oleifera* seed oil by SCF-CO2 and analysis of its constitution. China Oils and Fats. (2010) 35:76–9.

[32] Lin H. Study on antioxidation activities of Chinese dwarf chetty seed oil. Sci Technol Food Ind. (2012) 33:105–7+111. 10.13386/j.issn1002-0306.2012.15.010

[33] Giese EC Gascon J Anzelmo G Barbosa AM da Cunha MAA Dekker RFH. Free-radical scavenging properties and antioxidant activities of botryosphaeran and some other β-D-glucans. Int J Biol Macromol. (2015) 72:125–30. 10.1016/j.ijbiomac.2014.07.046 25128096

[34] Qiu X Wang Y Dong M Zhou J. Effect of enzymatic hydrolysis on juice yield and total antioxidant capacity in blueberry. Food Sci. (2013) 34:25–9. 10.7506/spkx1002-6630-201324005

[35] Lin X Ye H Xu Q Xu Z. Optimization for supercritical CO2 extraction of semen alli tuberosi oil by response surface methodology. J Chin Cereals Oils Assoc. (2021) 36:1–8. 10.3969/j.issn.1003-0174.2021.08.009

[36] Nogoy KMC Kim HJ Lee Y Zhang Y Yu J Lee DH. High dietary oleic acid in olive oil-supplemented diet enhanced omega-3 fatty acid in blood plasma of rats. Food Sci Nutr. (2020) 8:3617–25. 10.1002/fsn3.1644 32724624 PMC7382191

[37] Estruch R Ros E Salas-Salvadó J Covas M-I Corella D Arós F. Primary prevention of cardiovascular disease with a mediterranean diet supplemented with extra-virgin olive oil or nuts. N E J Med. (2018) 378:e34. 10.1056/NEJMoa1800389 29897866

