# Supplementary material for: Optimization of Supercritical CO2 Extraction of Moringa oleifera Seed Oil Using Response Surface Methodological Approach and Its Antioxidant Activity
Source: Front Nutr. 2022 Jan 21;8:829146. doi: 10.3389/fnut.2021.829146 (PMC8814622; doi:10.3389/fnut.2021.829146)
Supplement: Supplementary file 1 [file Table_1.DOCX]

**Table S1.** Comparison of DPPH scavenging ability of different edible oils and vitamin E

| Sample | EC_50_ |
| --- | --- |
| Moringa seed oil | 22.94 mg/mL |
| Peanut oil | 29.98 mg/mL |
| Tea oil | 8.39 mg/mL |
| Vitamin E | 3.45 mg/L |

**Table S2.** Comparison of hydroxyl radical scavenging ability of different edible oils and vitamin E

| Sample | EC_50_ |
| --- | --- |
| Moringa seed oil | 1.21 mg/mL |
| Peanut oil | 5.71 mg/mL |
| Tea oil | 2.28 mg/mL |
| Vitamin E | 70.5 mg/L |

**Table S3.** Comparison of average inhibition rate and maximum inhibition rate of different edible oils and vitamin E

| Sample | Average inhibition rate (%) | Maximum inhibition rate (%) |
| --- | --- | --- |
| Moringa seed oil | 81.0 | 97 |
| Peanut oil | 64.4 | 65.7 |
| Tea oil | 75.7 | 84.7 |
| Vitamin E | 64.0 | 98.1 |
